# A Study on the Liquid Helium Temperature Tensile Property of Fe-21Cr-15Ni-5Mn-2Mo Austenitic Stainless Steel after Solution Treatment

**DOI:** 10.3390/ma17184597

**Published:** 2024-09-19

**Authors:** Mengxing Zhang, Changjun Wang, Dangshen Ma, Yu Liu, Weijun Wang, Jianxiong Liang, Chao Fang, Weihan Chu, Chuanjun Huang

**Affiliations:** 1 Special Steel Research Institute, Central Iron and Steel Research Institute Co., Ltd., Beijing 100081, China; 15896513330@163.com (M.Z.); liuyu@nercast.com (Y.L.); liangjianxiong@nercast.com (J.L.); chuweihan@nercast.com (W.C.); 2 Hefei Institutes of Physical Science, Chinese Academy of Sciences, Hefei 230031, China; weijun.wang@ipp.ac.cn (W.W.); fangchao@ipp.ac.cn (C.F.); 3 Key Laboratory of Cryogenics, Technical Institute of Physics and Chemistry, Chinese Academy of Sciences, Beijing 100190, China; cjhuang@mail.ipc.ac.cn

**Keywords:** non-magnetic austenitic stainless steel, solution treatment, microstructure, 4.2 K tensile property, deformation twins

## Abstract

A novel non-magnetic Fe-21Cr-15Ni-5Mn-2Mo austenitic stainless steel with high strength and plasticity has been developed. The microstructure and liquid helium temperature (4.2 K) tensile properties of the top and bottom samples of large-size forged flat steel after solution treatment at 1090 °C were investigated. The results showed that the average grain size of the bottom sample (48.0 ± 6.7 μm) was smaller than that of the top sample (58.8 ± 15.3 μm), and the MX precipitates and Z phases were distributed in the matrix of the samples. The 4.2 K strengths of the samples at the top and bottom were high, and large amounts of annealing twin boundaries played a certain role in strengthening. After cryogenic tensile testing, large amounts of deformation twins, stacking faults, and dislocations were generated inside the austenite grains of both samples, which helped the material to obtain higher plasticity and strength. The top and bottom samples possessed excellent synergies of strength and plasticity at 4.2 K, and the 4.2 K tensile properties of the top sample were as follows: ultimate tensile strength (UTS) of 1850 MPa, yield strength (YS) of 1363 MPa, and elongation (EL) of 26%. The tested steel is thus believed to meet the requirements of combined excellent strength and plasticity within a deep cryogenic environment, and it would be a promising material candidate for manufacturing superconducting coil cases to serve in new generation fusion engineering.

## 1. Introduction

As fusion engineering advances, the performance requirements for large-scale ultra-low temperature structural materials used in fusion magnet systems are increasing rapidly. Austenitic steels are the preferred structural materials for manufacturing high-field superconducting magnets. Especially at 4.2 K, nitrogen-strengthened austenitic stainless type 316LN is widely used due to its thermodynamic stability and high strength [1,2]. In addition, in the 1980s, JJ1 (0.03C-12Cr-12Ni-10Mn-5Mo-0.2N) and JKBL (22Mn-13Cr-9Ni-1Mo-0.24 N) steels independently developed by Japan were also designed for superconducting magnets in the Fusion Experimental Reactor [3,4]. The China Fusion Engineering Test Reactor (CFETR) is the next step in Chinese-designed nuclear fusion, a bridge between the International Thermonuclear Experimental Reactor (ITER) and the Demonstration Power Plant (DEMO) [5,6].

Compared to ITER’s 11.8 T and 68 kA operating currents, the multiple CFETR TF magnet will provide a larger rated peak field of 14.5 T and 95.6 kA operating currents and is one of the most important components of the Tokamak magnet system [7]. Due to the large electromagnetic force generated by the high magnetic field and current, the improved 316LN and JJ1 applied to ITER TF coil case materials (YS ≈ 1000 MPa at 4.2 K) [8,9,10] cannot meet the strength requirements of the CFETR TF coil case (YS ≥ 1250 MPa and EL ≥ 25% at 4.2 K). Therefore, investigating and developing stronger and tougher weldable alloys for cryogenic structural components is one of the urgent problems to be solved in the construction of CFETR. The service environment of structural materials for fusion engineering is a 4.2 K ultra-low temperature and a 14.5 T ultra-strong magnetic field, which requires structural materials to have the characteristics of non-magnetism, ultra-high strength–toughness, and ultra-high strength–plasticity at 4.2 K. Based on this, our team developed a novel nitrogen-strengthening non-magnetic austenitic stainless steel Fe-21Cr-15Ni-5Mn-2Mo with high strength–toughness for fusion engineering through alloy composition design. During the production process of non-magnetic austenitic stainless steel for the large-size coil case structure, solution treatment is generally used after forging to obtain higher plasticity–toughness, high corrosion resistance, and low magnetic permeability.

So far, extensive studies have been carried out on structural materials for future fusion engineering. For example, according to Xin [11] et al., Fe-Cr-Ni-Mn-N austenitic steel exhibits outstanding cryogenic strength and an ultra-high strain hardening rate due to the formation of large amounts of stacking faults, deformation twins, and dislocations in the 4.2 K tensile test. Kato et al. [1] evaluated the cryogenic mechanical properties of XM-19 austenitic stainless steel with different specifications and sampling positions after solution treatment at 800 °C to 1200 °C, and found that the elongation at the center was low due to the segregation of the Nb element in the center of the 100 mm thick plate. Nishimura et al. [12] reported the 4.2 K tensile properties of XM-19 plates after composition optimization, and also found that the yield strength and elongation of the 100 mm thick plate were low due to the segregation of the Nb element. They believed that the yield strength and elongation of the middle part of the large-size forgings could be improved by optimizing the chemical composition and solution heat treatment. Due to the large scale of the structural parts used in fusion engineering, it is necessary to supply ultra-thick plates or forgings.

As mentioned above, although many researchers have paid attention to some related structure materials, it is worth noting that these studies mainly focus on test pieces with a thickness of about 100 mm, and the main research objects are XM-19 series steels. However, there is scarce insight into the overall performance of the ultra-low temperature mechanical properties of full-size coil case forging (the thickness is about 290 mm) for novel non-magnetic austenitic stainless steel. Therefore, the microstructural evolution and the ultra-low temperature tensile properties of samples taken from the central position of the top and bottom of full-size experimental steel have been studied in this work. The objectives of this work are to analyze the microstructures, the ultra-low temperature mechanical properties, and the corresponding deformation mechanism of samples taken from the central position of the top and bottom of experimental steel after 4.2 K tensile testing following solution treatment at 1090 °C, and to evaluate their performance as a new generation of structural materials for fusion engineering.

## 2. Experimental Procedure

### 2.1. Material Preparation and Heat Treatment

Fe-21Cr-15Ni-5Mn-2Mo steel has a high nitrogen content and very low oxygen and hydrogen content. In addition, its carbon content also requires lower levels for better performance, with a large (7 tons) production size. These characteristics make experimental steel smelting by electroslag remelting possible (ESR) [13]. The steel was forged into a large-size flat material with a size of 290 mm × 560 mm × 5000 mm, which was solution-treated at 1090 °C for 6 h after forging and then water-quenched to room temperature. The chemical composition of the experimental steel is shown in Table 1. The majority of elemental compositions are measured using the ICP-MS method with a plasma MS 300 instrument (NCS Testing Technology Co., Ltd. (NCS), Beijing, China)). Additionally, the nitrogen (N) element is determined using an N5500 nitrogen analyzer (NCS, Beijing, China), while the carbon (C) element and the sulfur element are analyzed with a CS3500G carbon and sulfur analyzer.

### 2.2. Microstructure and Mechanical Property Characterization

**Figure 1:**
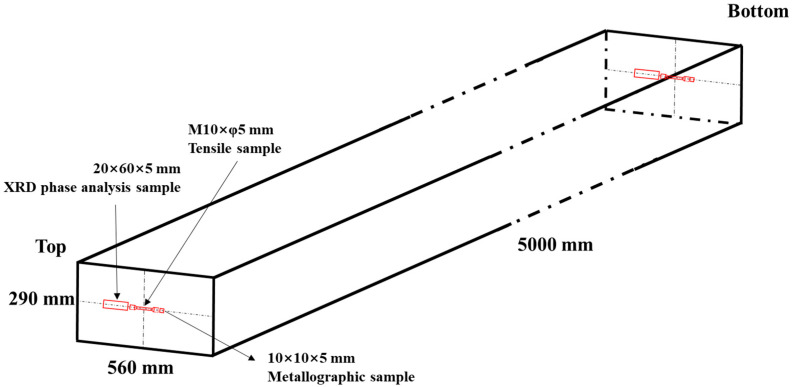
Schematic illustration of test specimens taken from prototype steel forging.

Metallographic specimens with 10 mm × 10 mm × 5 mm, XRD phase analysis specimens with 20 mm × 60 mm × 5 mm, and tensile specimens with φ5 mm × 65 mm (gauge length of 25 mm) were taken from the central positions of the top and bottom of the experimental steel, as shown in Figure 1. After grinding and polishing the surface, the samples were electrolyzed with chromic acid (chromic acid: 10 g, H_2_O: 100 mL) for 50–60 s to show the microstructures and etched with 10% potassium permanganate solution (H_2_SO_4_: 10 mL, H_2_O: 90 mL, KMnO_4_: 1 g) for 20–24 h to reveal grain sizes. The average grain sizes were measured by IPP software (version 6.0). The liquid helium temperature tensile tests were carried on the SUNS Model 5305S (Shenzhen SUNS Technology Stock Co., Ltd. Shenzhen, China), with a capacity of 300 kN and a tensile speed of 1.0 mm·min^−1^. The microhardness values of the samples were measured using a Vickers hardness tester EM500-2A (Hengyi precision instrument Co., Ltd., Shanghai, China) with a load of 300 g and a dwell time of 10 s. Ten indentations were randomly selected from the metallographic specimens. The plane for the hardness test is perpendicular to the forging direction. An optical microscope (OM) and FEI Quanta 650FEG scanning electron microscope (SEM, with an energy dispersive spectrum (EDS)) (Thermo Fisher Scientific, Waltham, MA, USA) were used to observe the microstructure of the specimens, and the EDS was used to analyze the compositions of the precipitated phases in the samples. The crystal phase distribution of the samples was analyzed by electron backscattering diffraction (EBSD, Symmetry S3, JEOL Ltd. Tokyo, Japan). The scanning step in the detection of EBSD was 0.3 μm. The preparation of the EBSD samples involved the use of a KClO_4_ + C_2_H_5_OH (1:9) solution to electropolish the polished small pieces. The polishing voltage was 30 V and the time was 25–30 s. The precipitated phase powder samples extracted from the matrix through electrolysis of the specimens were characterized by X-ray diffraction (XRD). The crystal structure and composition of the precipitated phase were further characterized by FEI Tecnai G^2^F20 transmission electron microscopy (TEM) (Thermo Fisher Scientific, Waltham, MA, USA). The TEM thin foils were prepared using a solution containing 10 vol % perchloric acid and 90 vol % ethanol at a temperature of −20–(−30 °C) and a current of 55 mA in a twin-jet electrolytic polishing device.

## 3. Results and Discussion

### 3.1. Microstructures

**Figure 2:**
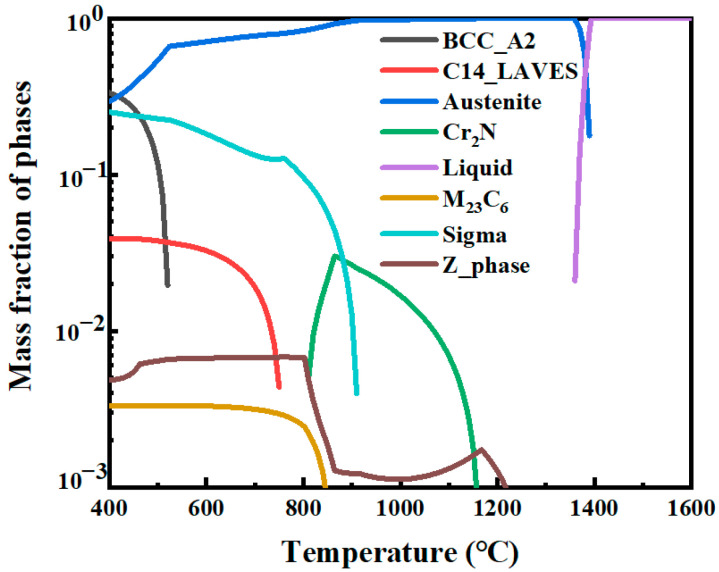
The equilibrium phase diagram.

**Figure 3:**
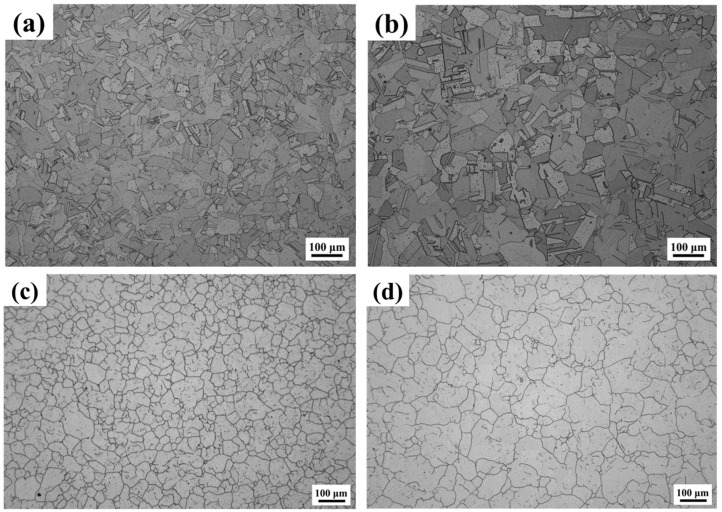
(**a**,**b**) Microstructures; (**c**,**d**) austenite grains of bottom sample (**a**,**c**) and top sample (**b**,**d**).

Figure 2 shows the equilibrium phase diagram of the experimental steel, which was calculated by using Thermo-Calc software (version 2021a) and the TCFE10 database. It can be seen that there are mainly an austenite matrix phase and two precipitated phases, a Cr_2_N phase and a Z phase (CrNbN), in the samples at 1090 °C. Figure 3 shows the microstructures of the top and bottom samples. Figure 3a,b indicate that the microstructure is composed of a single austenite with large amounts of annealing twins in both samples after solution treatment. Figure 3c,d show that the grain distribution in both samples is not uniform, which is a typical non-equiaxial grain. The average sizes of the samples are 58.8 ± 15.3 μm (top) and 48.0 ± 6.7 μm (bottom), respectively, obtained through statistical analysis using the cut-point method of the 100-times metallographic grain size pictures under different fields of view. Under annealing conditions, in austenite steel, twin sheets with straight boundary planes are often found in the recrystallization structure, i.e., annealed twins, and the formation of annealing twins reduces the total interfacial energy [14]. Only when large numbers of annealing twins are obtained after heat treatment can large numbers of deformation twins be generated in the subsequent tensile test process; so, the number of annealing twins directly affects the mechanical properties of the material [15].

**Figure 4:**
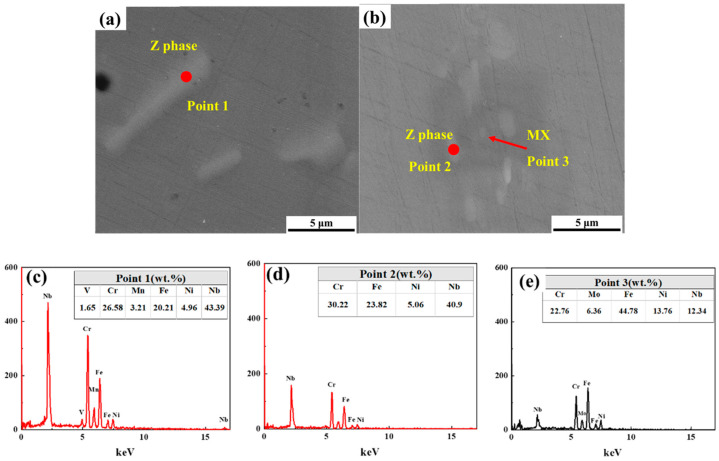
Precipitated phases observed using SEM. (**a**,**b**) Z phase and MX phase; (**c**–**e**) corresponding EDS of bottom sample (**a**,**c**) and top sample (**b**,**d**,**e**).

It can be seen from Figure 4a,b that large amounts of precipitated phases of different sizes are randomly distributed in the matrix in both samples. The EDS results (Figure 4c–e) show that there are two types of precipitated phases in the samples, which are mainly composed of Nb, Cr, Fe, and Ni elements. One type of precipitated phase shows that the content of the Nb element is the highest, and it can be determined that the precipitated phase is the Z phase (Point 1 and Point 2) (Figure 4a–d) through subsequent studies. In addition, there is the MX phase (Nb(C, N)) (Point 3) (Figure 4b,e), which is a relatively small-size phase. Compared with the Z phase, it contains a certain amount of Nb, but the content of Fe is the highest, and the content of Ni is also higher. These Nb-containing phases cannot be completely dissolved in the matrix after solid-solution treatment, which is consistent with the previous results of austenitic stainless steel with Nb added [16,17].

### 3.2. Precipitated Phase Analysis

**Figure 5:**
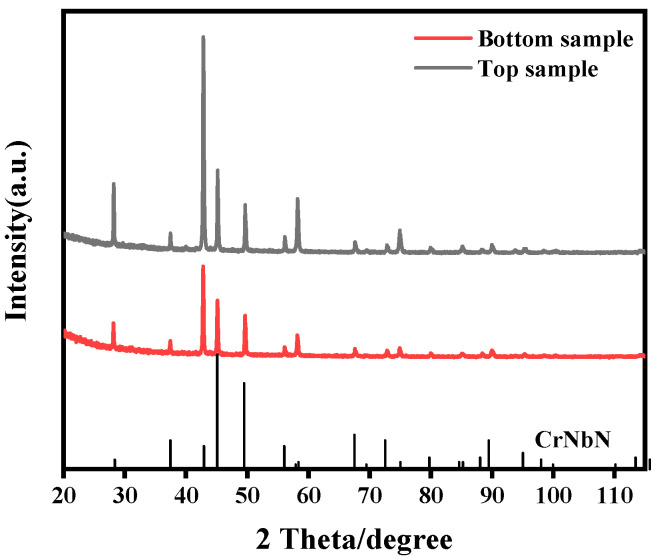
Extracted powder XRD results of bottom sample and top sample.

As shown in Figure 5, the XRD results indicate that only a set of peaks of the Z phase with a simple lattice tetragonal system can be detected from the powders in the top and bottom samples. No diffraction peak of the MX phase was found in both samples, which is different from the SEM results (Figure 4). The reasons why MX was not found may be that the size of MX was small, the content was less, and during the extraction process of the XRD test, due to the similar composition of the MX phase and the Z phase, the MX and Z phases may be mixed, resulting in the detection of only the Z phase.

**Figure 6:**
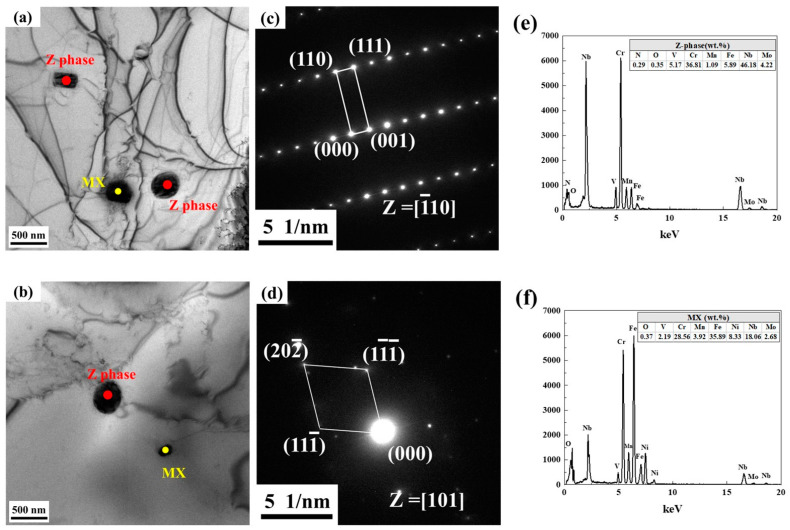
TEM observation. (**a**) Z phase and MX phase in bottom sample; (**b**) Z phase and MX phase in top sample; (**c**–**f**) SAED and EDS of Z phase and MX phase; (**c**,**e**) Z phase and (**d**,**f**) MX (NbN).

As shown in Figure 6a,b, the TEM results showed that there were long rods and nearly circular precipitates distributed in the crystal in the matrix of the top and bottom samples. According to the phase diagram (Figure 2), XRD results (Figure 5), and diffraction points (Figure 6c,d), it is determined that there are two phases in the matrix of the two samples, namely the Z phase (mainly composed of Nb, Cr, Fe, N elements, etc.) (Figure 6e) and the MX phase (mainly composed of Fe, Cr, Nb, Ni) (Figure 6f). As can be seen from Figure 6e,f, the Z phase does not contain the Ni element, and the contents of Nb and Cr are high. The MX phase contains a small amount of Ni and a high content of Fe. Different from the EDS results of the SEM, the Z phase components detected by EDS in TEM contain no Ni element. However, the Z phase in Figure 4c,d contains a small amount of the Ni element, which may be caused by the inaccuracy of the EDS or the spot hitting the matrix.

The Z phase in Figure 6c has a tetragonal crystal structure with lattice constants of a = b = 3.037 Å, c = 7.391 Å, which is consistent with the Z phase structure identified by Jack et al. [18]. The MX in Figure 6d is NbN with a face-centered cubic structure and a lattice constant of a = b = c = 4.3927 Å. Li et al. [19] believe that Z-complementary nucleation is related to the precipitation of NbN, and the dissolution of NbN will provide Nb, Cr, and N elements required for the growth of the Z phase. In addition, it has been reported that the Z phase can be precipitated directly in the matrix [20]. In this study, NbN precipitated phases were found in the top and bottom samples, and the size of the Z phase varied from large to small; so, the Z phase in the samples may be transformed by NbN or may be precipitated directly from the matrix.

### 3.3. 4.2 K Tensile Properties and Room Temperature Microhardness

**Figure 7:**
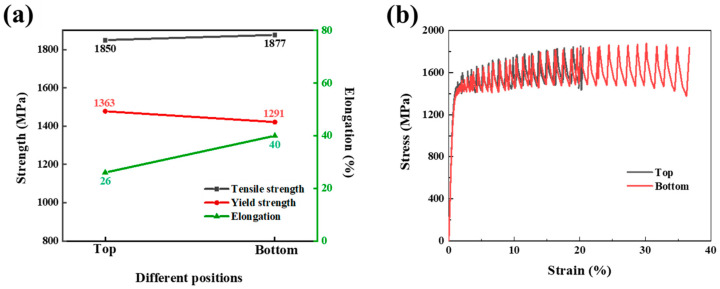
(**a**) Tensile properties at 4.2 K of experimental steel; (**b**) stress–strain curves.

Figure 7 illustrates the tensile properties and stress–strain curves of the top and bottom specimens at liquid helium temperature. It is evident that at the test temperature of 4.2 K, both specimens exhibit high strength, with ultimate tensile strengths (UTSs) exceeding 1850 MPa and yield strengths (YSs) greater than 1290 MPa. The elongation (EL) of the bottom sample is 40%, whereas the top sample, although slightly lower, still demonstrates a high level of plasticity, with an elongation of 26%. Based on the tensile test outcomes, the novel experimental steel is characterized by its high strength and plasticity.

**Figure 8:**
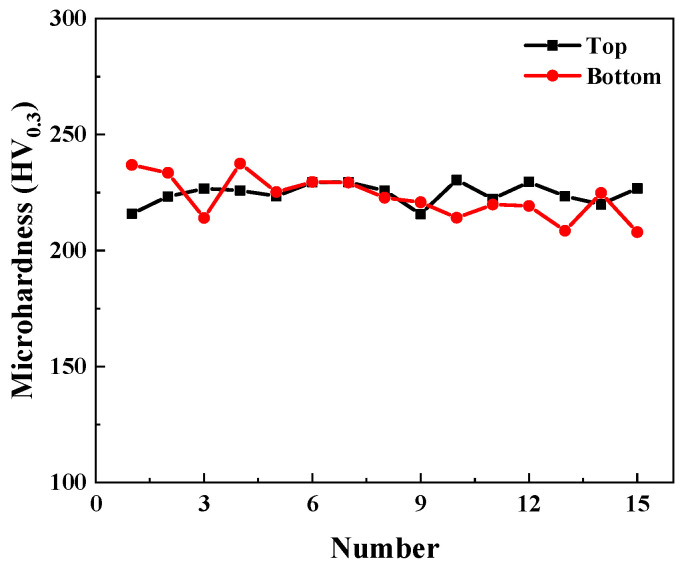
Microhardness of top and bottom samples.

Figure 8 shows the microhardness values of the top and bottom samples. It can be seen that the average microhardness of the top sample is about 224.5 ± 4.7 HV_0.3_. And the average microhardness of the bottom sample is 222.9 ± 9.4 HV_0.3_. The microhardness values of the top sample are more evenly distributed, but the hardness values between the top and bottom samples show little variation.

**Figure 9:**
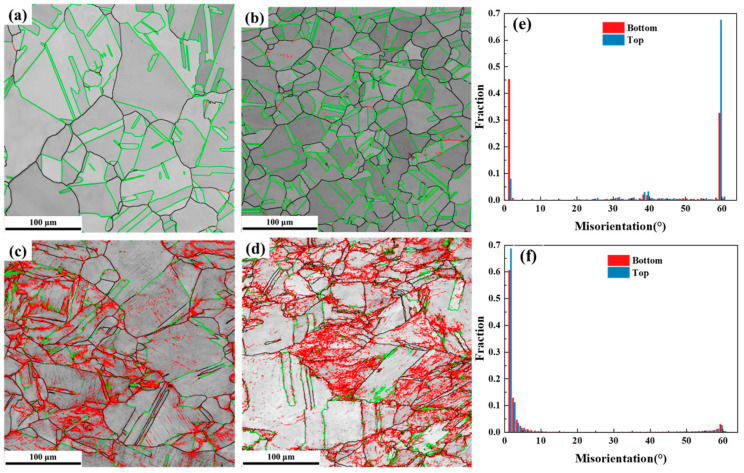
EBSD analyses of samples before (**a**,**b**,**e**) and after (**c**,**d**,**f**) tensile fracture of top (**a**,**c**) and bottom (**b**,**d**) samples and misorientation distributions (**e**,**f**). Low angle grain boundaries, high angle grain boundaries and twinning grain boundaries are respectively shown in red, black and green lines in (**a**–**d**).

In this work, Fe-21Cr-15Ni-5Mn-2Mo non-magnetic austenitic stainless steel is an FCC crystal system, its twin system is {111} <112>, and the twin grain boundary and the matrix meet the rotating axis relationship of 60° <111>, i.e., the matrix rotates 60° along the <111> axis [21]. The grain boundary can hinder the dislocation movement under the action of applied stress, resulting in stress concentration and many defects at the grain boundary, so the twins will preferentially nucleate at the grain boundary [21]. Figure 9 shows the microstructure and local misorientation distribution of the bottom and top samples before and after tensile deformation. In Figure 9a–d, the black lines are the high-angled grain boundaries of >15°, the red lines are the low-angled grain boundaries from 2° to 15°, and the green lines are the 60° <111> twinning grain boundaries (Σ3 grain boundaries). It can also be seen from Figure 9c,d that the red lines are relatively messy, and most of them do not have complete grain boundary characteristics, which may be caused by the excessively large scanning step size (0.3 μm) selected during the EBSD test.

Figure 9e shows that there are fewer low-angled grain boundaries between 2° and 10° in the specimens before tensile deformation, and the low-angled grain boundaries in the bottom sample are relatively high, which may be caused by more non-metallic inclusions in the bottom sample (Figure 9b). Furthermore, there are more high-angled grain boundaries of about 60° in the bottom and top samples before deformation, which should be caused by large numbers of annealing twins in the samples after solution treatment (Figure 3a,b). It can be seen from Figure 9f that the local misorientation distribution in both the bottom and top samples presents double peaks after tensile deformation, which has large numbers of low-angled grain boundaries and partial Σ3 grain boundaries. By comparing the local misorientation distribution in the samples before deformation (Figure 9e), it can be found that the low-angled grain boundaries in the range of 2°–10 ° increase significantly after tensile deformation, and the high-angled grain boundaries of about 60° dramatically decrease but still exist. The annealing twin boundaries near 60° after the tensile test are significantly reduced, indicating that the annealing twins make a great contribution to the tensile deformation. The higher content of annealed twin boundaries in the top specimen compared to the bottom specimen leads to stress concentration, resulting in a higher yield strength and lower elongation. Despite this, the top sample still maintains a high level of plasticity.

## 4. Discussion

**Figure 10:**
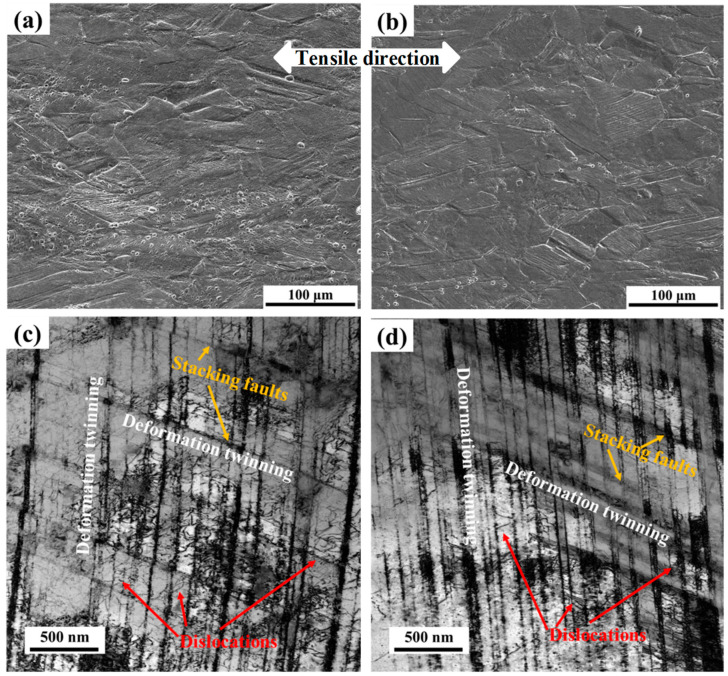
Deformed microstructures after tensile test using SEM (**a**,**b**) and TEM (**c**,**d**) of top samples (**a**,**c**) and bottom samples (**b**,**d**).

The microstructures of the longitudinal section samples near the fracture after 4.2 K tensile testing are shown in Figure 10. It can be seen from Figure 10a,b that deformation micro-bands exist in part of the austenite grains and the austenite grains are elongated along the deformation direction. It can be observed from Figure 10c,d that after 4.2 K tensile testing, both the top and bottom samples exhibit large amounts of deformation twins, stacking faults, and dislocations. Consequently, those deformation micro-bands may be deformation twins.

The grain boundary structure and properties of polycrystalline metal materials have an important influence on the mechanical properties of the materials [22,23]. Wang et al. [24] explained that the twin boundaries generated after solution treatment of TWIP steel are beneficial for increasing other special grain boundaries (low Σ-CSL grain boundaries). Large numbers of special grain boundaries, including twin grain boundaries, can effectively destroy the connectivity of random grain boundaries, which can reduce the stress concentration near the grain boundaries and is conducive to improving the plasticity of the material. Under these experiment conditions, the number of annealing twins in the bottom and top samples is large, and the generation of annealing twin boundaries is conducive to increasing the number of other special grain boundaries and effectively breaking the connectivity of random grain boundaries, so that the cracks cannot continue to propagate along the random grain boundaries in the process of deformation, which is macroscopically manifested as the increase in elongation during tensile stretching.

It can be seen from Figure 10a,b that a small number of initial annealing twins can also be observed in the deformed microstructure, which is consistent with Figure 9c,d,f, indicating that most of the annealing twins are involved in the deformation. The deformation twins penetrate throughout the austenite grain from the grain boundary, and the austenite grains are segmented. The fine deformation twins are interlaced and distributed in the austenite grains, which play the role of subgrain boundaries, refine the matrix structure, and make a certain contribution to the strength. This is achieved by continuous grain fragmentation caused by the introduction of new interfaces, effectively reducing the mean free path of dislocations and leading to strengthening [25,26,27,28,29]. This phenomenon is often referred to as the ‘dynamic Hall–Petch’ effect.

A single austenite accompanied by large numbers of annealing twins is the microstructure of experimental steel. After the 4.2 K tensile test, large amounts of deformation twins, stacking faults, and dislocations are generated in the austenite grains. The deformation twin grain boundary essentially plays the role of a subgrain boundary and refines the matrix to some degree, i.e., ‘dynamic Hall–Petch’ effect, which makes a certain contribution to the strength. The twinning deformation provides the possibility for the movement of a slip system with unfavorable orientation or one that is difficult to slip by changing the crystal orientation, which is more conducive to the uniform deformation of the matrix metal, and the twin itself also has a certain plastic deformation; so, the twining deformation greatly improves the elongation after the break. Therefore, the novel developed experimental steel has excellent combined strength and plasticity/elongation.

## 5. Conclusions

In this study, Fe-21Cr-15Ni-5Mn-2Mo non-magnetic austenitic stainless steel with a size of 290 mm × 560 mm × L mm was heat-treated with 1090 °C solution temperatures. The microstructures, the ultra-low temperature mechanical properties, and the corresponding deformation mechanism of samples after 4.2 K tensile testing were studied; the main conclusions are as follows:

(1) After solution treatment at 1090 °C for 6 h and then being water-quenched to room temperature, the microstructure is composed of a single austenite with large numbers of annealing twins in both the top and bottom samples of the experimental steel, and the grain size of the top sample (58.8 μm) is larger than that of the bottom sample (48.0 μm). There are two precipitated phases in both samples, i.e., the Z phase and the MX phase.
(2) Large amounts of deformation twins, stacking faults, and dislocations appeared in the 4.2 K tensile specimens, which is beneficial for improving strength and elongation. The “dynamic Hall–Petch” effect in the 4.2 K tensile process led to twin-induced strengthening.
(3) The novel developed experimental steel exhibits an excellent combination of strength and plasticity, and the properties of the top sample after 4.2 K tensile testing are as follows: the UTS, YS, and EL values are 1850 MPa, 1363 MPa, and 26%, respectively.

The experimental steel, with its excellent combination of strength and ductility, is suitable for the structural components of next-generation fusion engineering coil cases. However, future requirements for coil case materials in stronger fusion engineering reactors will demand even higher yield strengths. In the future, we will delve deeper into the mechanisms of strengthening and toughening to further optimize the properties of the experimental steel, aiming to achieve a yield strength of over 1500 MPa at 4.2 K.

## Figures and Tables

**Table 1:** Chemical composition of experimental steel (wt. %).

C	Mn	Si	S	P	Cr	Ni	Mo	V	Nb	N
0.010	5.25	0.17	0.0025	0.0013	20.98	14.90	2.35	0.17	0.12	0.35

## Data Availability

The data will be made available on request.
